# Association mapping for broomrape resistance in sunflower

**DOI:** 10.3389/fpls.2022.1056231

**Published:** 2023-01-06

**Authors:** Álvaro Calderón-González, Begoña Pérez-Vich, Nicolas Pouilly, Marie-Claude Boniface, Johann Louarn, Leonardo Velasco, Stéphane Muños

**Affiliations:** ^1^ Instituto de Agricultura Sostenible, Consejo Superior de Investigaciones Científicas (IAS-CSIC), Córdoba, Spain; ^2^ Laboratoire des Interactions Plantes Microbes-Environnement (LIPME), Université de Toulouse, CNRS, INRAE, Castanet-Tolosan, France

**Keywords:** broomrape resistance, genome-wide association mapping (GWAS), candidate genes, *Orobanche cumana*, parasitic plants

## Abstract

**Introduction:**

Sunflower breeding for resistance to the parasitic plant sunflower broomrape (Orobanche cumana Wallr.) requires the identification of novel resistance genes. In this research, we conducted a genome-wide association study (GWAS) to identify QTLs associated with broomrape resistance.

**Methods:**

The marker-trait associations were examined across a germplasm set composed of 104 sunflower accessions. They were genotyped with a 600k AXIOM® genome-wide array and evaluated for resistance to three populations of the parasite with varying levels of virulence (races EFR, FGV, and GTK) in two environments.

**Results and Discussion:**

The analysis of the genetic structure of the germplasm set revealed the presence of two main groups. The application of optimized treatments based on the general linear model (GLM) and the mixed linear model (MLM) allowed the detection of 14 SNP markers significantly associated with broomrape resistance. The highest number of marker-trait associations were identified on chromosome 3, clustered in two different genomic regions of this chromosome. Other associations were identified on chromosomes 5, 10, 13, and 16. Candidate genes for the main genomic regions associated with broomrape resistance were studied and discussed. Particularly, two significant SNPs on chromosome 3 associated with races EFR and FGV were found at two tightly linked SWEET sugar transporter genes. The results of this study have confirmed the role of some QTL on resistance to sunflower broomrape and have revealed new ones that may play an important role in the development of durable resistance to this parasitic weed in sunflower.

## 1. Introduction

Sunflower broomrape (*Orobanche cumana* Wallr.) is one of the main biotic stresses affecting sunflower. This holoparasitic plant parasitizes sunflower roots causing devastating effects if resistant cultivars and/or herbicide treatments are not used (Cvejić et al., 2020). The parasite has been traditionally present in many sunflower-producing areas of Europe and Asia (Fernández-Martínez et al., 2015). It has recently started to be detected in African countries such as Tunisia (Amri et al., 2012) and Morocco (Nabloussi et al., 2018).

Broomrape control strategies in sunflower have largely focused on using resistant cultivars. Their development was possible due to the existence of good sources of resistance, especially in wild *Helianthus* species, with monogenic inheritance in most cases (Fernández-Martínez et al., 2015; Cvejić et al., 2020). Genetic resistance to broomrape was introduced into sunflower in the early breeding programs in the former USSR in the first years of the 20th century (Velasco et al., 2016). However, the introduction of new resistance sources was followed by the appearance of new physiological races of the parasite that overcame resistance (Fernández-Martínez et al., 2015). Thus far, eight broomrape races designated with letters from A to H have been reported based on their virulence on sunflower differential lines (Cvejić et al., 2020). However, the current distinction between broomrape races in the main infested areas remains unclear, as there is little information on the correspondence of races with the same name reported in different countries (Cvejić et al., 2020). Races D and E were predominant until the middle 1990s, and they were satisfactorily controlled by the resistance gene *Or_5_*, widely used in commercial hybrids. Populations overcoming *Or_5_* resistance were detected in 1995 in Spain (Alonso et al., 1996) and shortly after in Romania, Turkey, and several other countries (Skoric et al., 2010). Currently, it seems clear that races more virulent than E (races F, G, and H) are predominant in most sunflower-producing areas where this parasite is present (Cvejić et al., 2020).

Genetic resistance to broomrape in sunflower has been found in most cases to be controlled by vertical resistance mechanisms that follow a gene-for-gene interaction, in which a dominant gene for host resistance interacts with a dominant avirulence gene in the parasite (Rodríguez-Ojeda et al., 2013). The genetic control of broomrape resistance by a single dominant gene was first reported by Pogorletsky and Geshele (1976). Shortly after, Vranceanu et al. (1980) identified five differential lines that had accumulative resistance to broomrape races A to E, controlled by five dominant resistant genes named *Or_1_* to *Or_5_*, respectively. Several other studies confirmed monogenic dominant resistance to race E (Sukno et al., 1999; Lu et al., 2000; Pérez-Vich et al., 2004). One dominant gene has also been reported controlling races overcoming *Or_5_* resistance, such as *Or_6_* conferring resistance to race F from Romania (Pacureanu-Joita et al., 2004), *Or_7_* controlling race F from Spain (Duriez et al., 2019), *Or_Deb2_* for resistance to race G from Turkey (G_TK_) (Velasco et al., 2012), and *Or_SII_* and *Or_Pra1_* providing posthaustorial resistance to races F and G (Sayago et al., 2018; Martín-Sanz et al., 2020). Several major dominant genes have been located on the sunflower genetic map. *Or_5_* has been mapped to a telomeric region of chromosome (chr) 3 (Lu et al., 2000; Tang et al., 2003; Pérez-Vich et al., 2004). Later, Imerovski et al. (2013) and Imerovski et al., (2016) found simple sequence repeat (SSR) markers of chr 3 strongly associated with resistance genes other than *Or_5_* such as *Or_2_*, *Or_3_*, and *Or_6_*. Recently, Duriez et al. (2019) have mapped *Or_7_* to chr 7, and Martín-Sanz et al. (2020) and Fernández-Aparicio et al. (2022) have located *Or_SII_* and *Or_Deb2_*, respectively, to the upper half of chr 4.

In addition to the studies on vertical resistance, molecular studies have also focused on more complex genetic systems influencing broomrape resistance in sunflower, e.g., quantitative trait loci (QTLs) that contribute with small-to-moderate effects to decreasing the number of emerged broomrapes (Pérez-Vich et al., 2004; Akhtouch et al., 2016; Imerovski et al., 2019). It has been demonstrated that resistance QTL may act at different broomrape developmental stages, providing accumulative resistance mechanisms (Louarn et al., 2016). Within this quantitative component, the role of (i) “defeated resistance genes” corresponding to major resistance genes specific for a broomrape race which provide only moderate levels of resistance to a different-more virulent race (Imerovski et al., 2019), and (ii) resistance QTL present in susceptible cultivars (Pérez-Vich et al., 2004; Akhtouch et al., 2016), have also been demonstrated. The combination of major resistance genes with quantitative resistance factors is seen as a promising alternative to ensure durable sunflower protection against *O. cumana* (Pérez-Vich et al., 2013).

Genome wide association study (GWAS) is a powerful tool to identify QTLs by examining the marker-trait associations across diverse germplasms. Compared to traditional genetic linkage analysis based on bi-parent populations, GWAS increases mapping resolution, reduces research time, and includes more alleles (Zhu et al., 2008). The availability of high-density SNP genotyping data, linkage maps, and the full genome sequence (Badouin et al., 2017), together with sufficient linkage disequilibrium (LD) decay (Kolkman et al., 2007), have made it feasible to carry out large scale GWAS in sunflower. Association mapping studies in this crop have focused on flowering time (Cadic et al., 2013; Mandel et al., 2013; Bonnafous et al., 2018), branching pattern (Mandel et al., 2013; Nambeesan et al., 2015), fertility restoration (Goryunov et al., 2019; Talukder et al., 2019) and floral traits (Dowell et al., 2019). However, very few studies have been conducted on disease resistance, all of them on fungal pathogens (Fusari et al., 2012; Talukder et al., 2014). No association mapping studies have been reported so far on *O. cumana* resistance. Such studies are very limited for resistance to other parasitic plant species, mainly centered on the interaction between *Striga hermonthica* and cereal crops (Adewale et al., 2020; Kavuluko et al, 2021).

In this study, we have used GWAS on a population of 104 diverse sunflower accessions with varying levels of resistance to *O. cumana*. The accessions were genotyped using a sunflower Affymetrix AXIOM Genome-Wide array and evaluated for resistance to three populations of *O. cumana* with varying levels of virulence in two environments for each broomrape population. The main objective of the study was to detect loci associated with resistance to this parasitic weed and to identify resistance candidate genes.

## 2. Materials and methods

### 2.1. Sunflower germplasm

The sunflower germplasm set included 104 accessions (**Table S1**) selected from the germplasm collections of the USDA-ARS (38), INRAE (46), and IAS-CSIC (20). Most of the accessions are of the oilseed type, although some of them are of the confectionery type. This information is provided in **Table S1**. Around one-third of the accessions (34) were selected because we had the previous indication that they possessed non-dominant resistance against broomrape, particularly to race F, but also in some cases to populations of race G. In general, the resistance of these lines was incomplete, i.e., they showed reduced infection but not immunity like the germplasm with dominant, vertical resistance. They were, in most cases, unpublished material, but some of the accessions have been reported previously, e.g., L86, K96, P96 and R96 (Fernández-Martínez et al., 2004), AM1, AM2 and AM3 (Pérez-Vich et al., 2006), and LR1 (Louarn et al., 2016).

### 2.2. Sunflower broomrape populations

Resistance of the sunflower accessions was evaluated with three contrasting sunflower broomrape populations from different origins and degrees of virulence. SP is a population of race F_GV_ from the Guadalquivir Valley (GV) collected in Écija, Andalusia, Spain. Bourret is a population of race E_FR_ collected in Bourret (Tarn et Garonne), Occitania, France. GT is a population belonging to race G_TK_ collected in Çeşmekolu, Thrace, Turkey. Broomrape nomenclature follows Martín-Sanz et al. (2016).

### 2.3. Evaluation of broomrape parasitism

Sunflower accessions were evaluated for their reaction to broomrape populations Bourret and GT in pots in 2016 and 2017 in Córdoba, Spain. For population SP, evaluation was conducted in pots in 2017 and the field in 2018 in the same location. SP population, which belongs to the race F_GV_ widely distributed in the area of the experiments, was the only population that could be evaluated under field conditions without the risk of introducing foreign populations in the area. In all cases, including the experiment in the field, all plants were inoculated with broomrape seeds as detailed below.

In all the experiments, sunflower seeds were germinated in moistened filter paper at 25 °C in the dark for 48 and sown in small pots 7 x7 x 7 cm filled with sand and peat and 50 mg of broomrape seeds. The soil mixture containing the broomrape seeds was shaken in a plastic bag to distribute broomrape seeds uniformly. The pots were maintained in a growth chamber at 25°C/20-°C (day/night) with 16 h photoperiod for six weeks, then transplanted into 5 L pots containing a soil mixture of sand, silt, and peat in a proportion 2:1:1 or to the field in the case of the field experiment in 2018. The pots were maintained under open-air conditions in the spring-summer period and watered as required. In the field, plants were watered with drip irrigation. Sowing dates were 9 to 11 March in 2016, 6 to 8 March in 2017, and 26 to 28 February in 2018. In pot experiments, seven pots per accession were used. In the field, the experiment included three replicates of eight plants each. In this case, the accessions were randomized within each replicate. Plant distance within the row was 33 cm, and the row separation was 1 m.

The number of emerged broomrape shoots (NEBS) was counted for each sunflower plant at the end of sunflower flowering.

Analysis of variance (ANOVA) was conducted on the number of emerged broomrape shoots using the accessions, the broomrape populations, and the environments (nested to the broomrape populations) as fixed factors. Mean squares values were used as an estimate of the relative weight of the factors on the number of emerged shoots. Pearson’s correlation coefficients were also computed between environments for a given broomrape population and between the two-year average NEBS value of the accessions for each broomrape population. Analyses were conducted using SPSS statistical package version 27.

### 2.4. Tissue collection, DNA extraction and plant genotyping

Genomic DNA for the 104 accessions was extracted from leaf tissue using the Kit DNeasy Plant Mini Kit (Qiagen^©^). The DNA concentration was adjusted to 10ng/μl in water. The genotyping experiments were performed by the Gentyane platform (Plateforme Gentyane, UMR INRAE/UBP 1095 Génétique Diversité et Ecophysiologie des Céréales, Clermont-Ferrand, France) on a GeneTitan^®^ (Affymetrix) according to the manufacturer’s instructions. The AXIOM array was built using a set of 586,985 SNPs. Genotypic data were obtained with the software Axiom Analysis Suite (http://www.affymetrix.com).

### 2.5. Genetic diversity and population structure analysis

The genotyping data were imputed by genetic linkage group using BEAGLE (Browning and Browning, 2009). We filtered genotyping data by keeping a single SNP when redundant to others, and we removed SNPs showing minor allele frequency (MAF < 5%). Final filtering was done with software TASSEL v5.2.59 (Bradbury et al., 2007), removing a total of twelve markers classified as unmapped, which were discarded to create the definitive set of markers used for subsequent analyses. The kinship matrix (K-matrix) was calculated using the Centered-IBS method on this set of high-quality filtered SNP markers. Finally, we kept a set of 23,743 SNPs for further analysis after removing redundant markers.

The analysis of the genetic structure and kinship patterns of the population was computed using STRUCTURE ver. 2.3.4 (Pritchard et al., 2000) using the set of 23,743 SNPs. An admixture model following the Hardy-Weinberg equilibrium was used. The analysis was repeated ten times for each value of K (from 1 to 10) using a burn-in period of 100,000 Markov Chain Monte Carlo (MCMC) iterations and a run length of 100,000. The number of groups in the population was determined using Structure Harvester (Earl and von Holdt, 2012) with the Evanno correction (Evanno et al., 2005). The output of STRUCTURE analysis was subjected to the FullSearch algorithm of CLUMPP ver. 1.1.2b (Jakobsson and Rosenberg, 2007), and the output was used to produce bar graphs of the population structure using Origin Pro 9.1 software (OriginLab Corporation, Northampton, MA, USA).

For the genetic diversity analysis, we used 6,264 SNP bi-allelic markers, i.e., one out of every fourth marker. Shannon’s information index (I) observed heterozygosity (Ho), Nei’s expected heterozygosity (He), and the fixation index (F) were computed. A principal coordinates analysis (PCoA) was also conducted. GenAlEx 6.501 (Peakall and Smouse, 2012) was used for these analyses.

### 2.6. Genome-wide association analysis and linkage disequilibrium

A panel of 23,743 SNP markers with MAF > 5% was used for GWAS. A preliminary analysis evaluated the performance of the general linear model (GLM) and the mixed linear model (MLM) using either the Q-matrix or PCA covariates as cofactors. Additionally, the kinship (K) matrix was added to the MLM models to avoid spurious associations linked to the genetic relatedness. For MLM models, we also tested several compression and variance component estimation options. The analyses were conducted using phenotypic data (average NEBS per sunflower plant) for each broomrape population and environment, and the average values for each broomrape population in the two environments. Quantile-quantile plots (QQ-plots) were constructed from the observed versus expected -log_10_(p) values of each model. The significance of marker-trait associations (MTAs) was checked based on Bonferroni and false discovery rate (FDR) corrections at 5% and 20% (Benjamini and Hochberg, 1995). Furthermore, the range of linkage disequilibrium (LD) was computed using a sliding window of 50 kbp. Manhattan-plots were generated with the position and the p-value. The analyses were performed using TASSEL software v. 5.2.56. The matrix of p-value was used to estimate the FDR with the QVALUE package (Storey, 2002) in R.

### 2.7. Candidate gene analyses

The significant marker-trait associations obtained were mapped on the HanXRQr2.0-SUNRISE reference sunflower genome sequence (https://www.heliagene.org/HanXRQr2.0-SUNRISE). After the physical positions were extracted, the genomic regions of the significant SNPs were examined to identify the annotated protein-coding genes located in or close to the significant SNPs. Exploration of the genomic regions for identification of candidate genes was carried out as follows: (i) if a cluster of significant marker-single trait associations was found, the SNP and the physical region spanned by the significant markers (+/- 250-Kb) was explored for high confidence genes with predicted biological function; and (ii) if only one single SNP marker constituted the significant marker-single trait association, the genes putatively involved in plant disease and parasitic plant-resistance pathways containing or immediately adjacent (within a window of 250 kb) to the SNPs were identified. Finally, if no candidate genes were found using these criteria, the closest candidate gene with known function in disease and parasitic plant-resistance pathways was also selected. The nature of most significant annotated candidate genes, and of all the genes coding for uncharacterized proteins, unknown function, or directly annotated but without description, was verified in the NCBI *Helianthus annuus* annotation release 101 (2020-09-02), and through BLAST searches using the sunflower sequences.

## 3. Results

### 3.1. Phenotypic evaluation of sunflower genotypes

The analysis of variance showed a marked effect of the environment on the number of emerged broomrape shoots, accounting for 67.3% of the total estimated variance (**Table 1**). It was followed by the broomrape population, which accounted for 28.4% of the variance, and the sunflower accession, which contributed with 3.3% to the total variance. These three main factors, as well as the interactions, were significant (P<0.01), although the interactions were of very low magnitude (**Table 1**).

**Table 1:** Analysis of variance for the number of emerged broomrape shoots in a set of 104 sunflower accessions evaluated for three broomrape populations in two environments for each population.

Source of variation	Degrees of freedom	Sum of squares	Mean squares	% MS	F	P
Accession	103	464458	4509	3.3	40.4	<0.01
Broomrape population	2	76607	38303	28.4	343.5	<0.01
Environment (Br. Population)	3	271712	90571	67.3	812.3	<0.01
Acession x Br. Population	206	109738	533	0.4	4.8	<0.01
Accession x Environment	308	190394	618	0.5	5.5	<0.01
Error	3359	374543	112	0.1		
Total	3982	3267402				

The average NEBS per sunflower plant ranged from 6.91 in the evaluation for broomrape population SP in 2018 to 36.3 for broomrape population GT in 2017 (**Table 2**). For the three broomrape populations, there was a variable number of sunflower accessions that showed a high degree of resistance. Considering the accessions that showed less than one broomrape shoot in the average of both evaluations, we observed 23 accessions for population SP, seven accessions for Bourret, and two accessions for GT. If we consider the six evaluations, two accessions showed less than one broomrape per plant (**Table S1**).

**Table 2:** Mean, standard deviation (SD), minimum and maximum values of emerged broomrape shoots in a set of 104 sunflower accessions evaluated for broomrape populations Bourret, SP and GT in two years.

Population/Year	Mean	SD	Minimum	Maximum
Bourret 2016	22.13	14.98	0.00	75.75
Bourret 2017	22.89	17.37	0.00	75.86
SP 2017	17.30	16.48	0.00	63.14
SP 2018	6.91	6.54	0.00	25.04
GT 2016	10.27	6.34	0.13	28.00
GT 2017	36.32	19.29	0.00	79.29

Despite the large influence of the environment on the NEBS, correlation coefficients between the evaluations for the same population in different environments or even for the evaluation of different populations in different environments were in all cases positive and statistically significant. Correlation coefficients between the two evaluations for each population ranged from 0.59 for population GT to 0.82 for population SP. Considering the correlation coefficients between different populations in individual environments, they ranged from 0.50 (GT in 2016 vs. Bourret in 2016) to 0.77 (Bourret in 2017 vs. SP in 2017), whereas the correlation coefficients between populations considering the average value of the two evaluations ranged from 0.72 (GT vs. Bourret) to 0.78 (Bourret vs. SP) (**Table 3**).

**Table 3:** Correlation coefficients between emerged broomrape shoots in a set of 104 sunflower accessions evaluated with three broomrape populations in two environments each one.

Evaluation	SP_2018	Bourret_2016	Bourret_2017	GT_2016	GT_2017	Bourret_Average	GT_Average
SP_2017	0.82**	0.66**	0.77**	0.59**	0.76**		
SP_2018		0.55**	0.63**	0.55**	0.65**		
Bourret_2016			0.67**	0.50**	0.61**		
Bourret_2017				0.51**	0.66**		
GT_2016					0.59**		
SP_Average						0.78**	0.78**
Bourret_Average							0.72**

Correlation coefficients between the average values in the two environments for each population are also included.

**Significant at p<0.01.

### 3.2. Genetic diversity, population structure and linkage disequilibrium analysis

Single nucleotide polymorphism (SNP) markers were evenly distributed across the whole genome, from 629 SNPs in chr 7 to 2605 SNPs in chr 8 (**Table 4**). The number of SNPs per Mbp ranged from 5.1 in chr 15 to 17.1 in chr 8.

**Table 4:** SNPs distribution across all the chromosomes with the position (bp) of the first and the last molecular marker per linkage group.

inkage group	Number of SNPs	Length (Mbp)	SNPs/Mbp	Physical position of the extreme markers in each chromosome (Mbp)
**1**	2045	153.3	13.3	0 (0.01) - 2044 (153.3)
**2**	1605	177.7	9.0	2045 (1.4) - 3649 (179.1)
**3**	1360	167.8	8.1	3650 (0.6) - 5009 (168.5)
**4**	1234	178.5	6.9	5010 (0.2) - 6243 (178.8)
**5**	2069	218.6	9.5	6244 (0.4) - 8312 (219.1)
**6**	703	102.5	6.9	8313 (1.0) - 9015 (103.5)
**7**	629	103.8	6.1	9016 (0.05) - 9644 (103.8)
**8**	2605	152.4	17.1	9645 (0.04) - 12249 (152.5)
**9**	1206	207.8	5.8	12250 (1.3) - 13455 (209.1)
**10**	2101	245.3	8.6	13456 (0.9) - 15556 (246.2)
**11**	1055	167.9	6.3	15557 (0.01) - 16611 (167.)
**12**	989	165.6	6.0	16612 (0.05) - 17600 (165.7)
**13**	1360	195.7	6.9	17601 (1.1) - 18960 (196.8)
**14**	1108	173.9	6.4	18961 (0.5) - 20068 (174.3)
**15**	874	169.8	5.1	20069 (1.4) - 20942 (171.2)
**16**	1976	187.8	10.5	20943 (0.7) - 22918 (188.5)
**17**	2027	214.7	9.4	22919 (0.01) - 24945 (214.7)

Genetic diversity analysis revealed that the means of the effective and observed allele numbers for the sunflower set were 2.0 and 1.75, respectively. The expected heterozygosity (Nei’s gene diversity) and Shannon’s information index were 0.42 and 0.60, respectively. The observed heterozygosity and the fixation index were 0.04 and 0.92, respectively.

**Figure 1:**
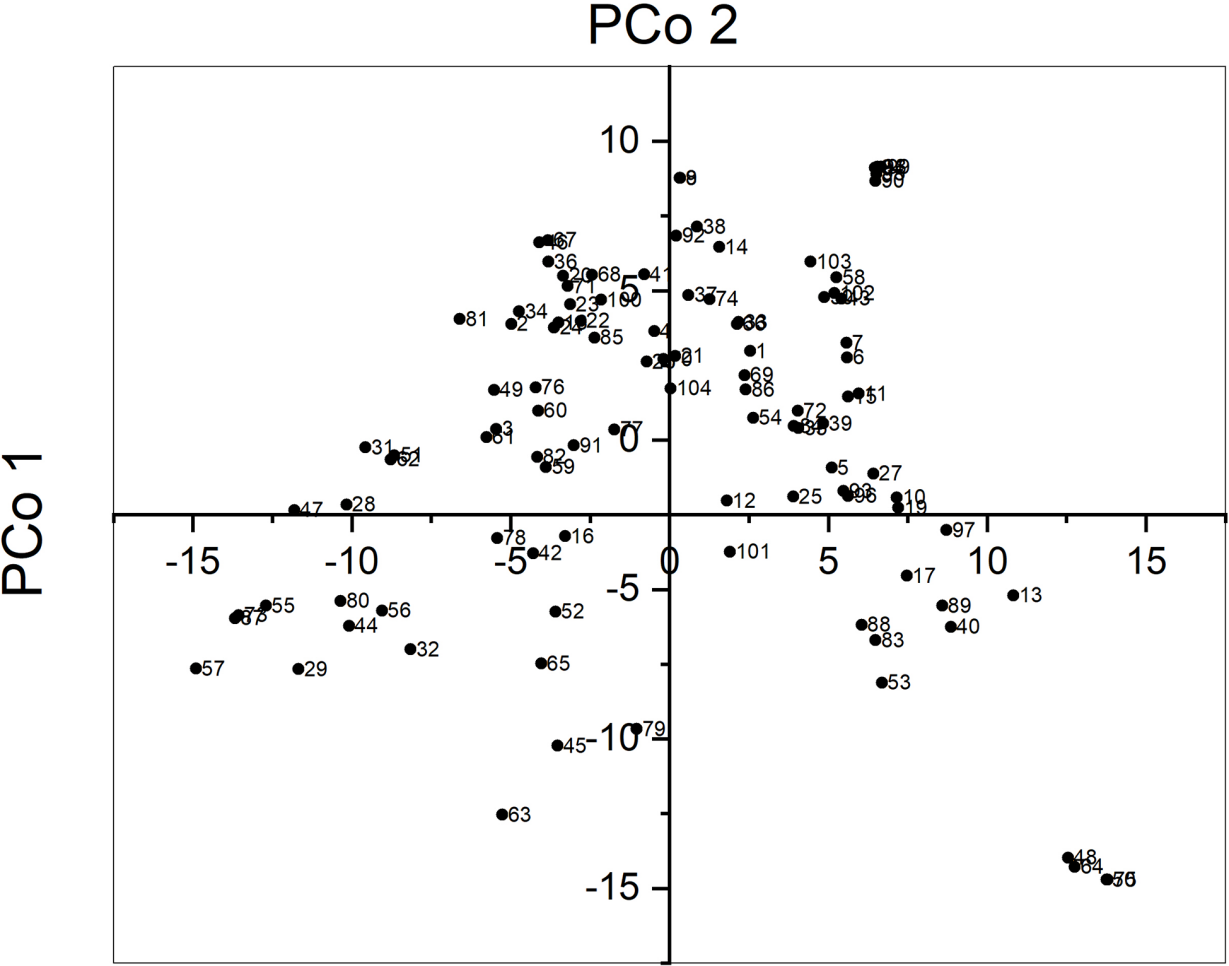
Principal coordinate analysis of 104 sunflower accessions genotypes with SNP markers.

The average pairwise genetic distance between sunflower accessions was 10,475 and ranged from 126 for accessions CD and HA89 to 13,423 for accessions UD and PI578010. Principal Coordinate Analysis (PCoA) revealed that the three first axes explained a low proportion of the total variance, 8.1, 6.4, and 4.3%, respectively. **Figure 1** shows the biplot for PCo 1 and PCo 2. Some accessions were grouped very closely, for example accessions PO7-28, PO7-34, PO7-38, PO7-61, and PO7-63. They were developed in a recurrent selection program starting from a random mating population with selection for broomrape race F (unpublished). Their relatedness was unknown at the beginning of the research.

**Figure 2:**
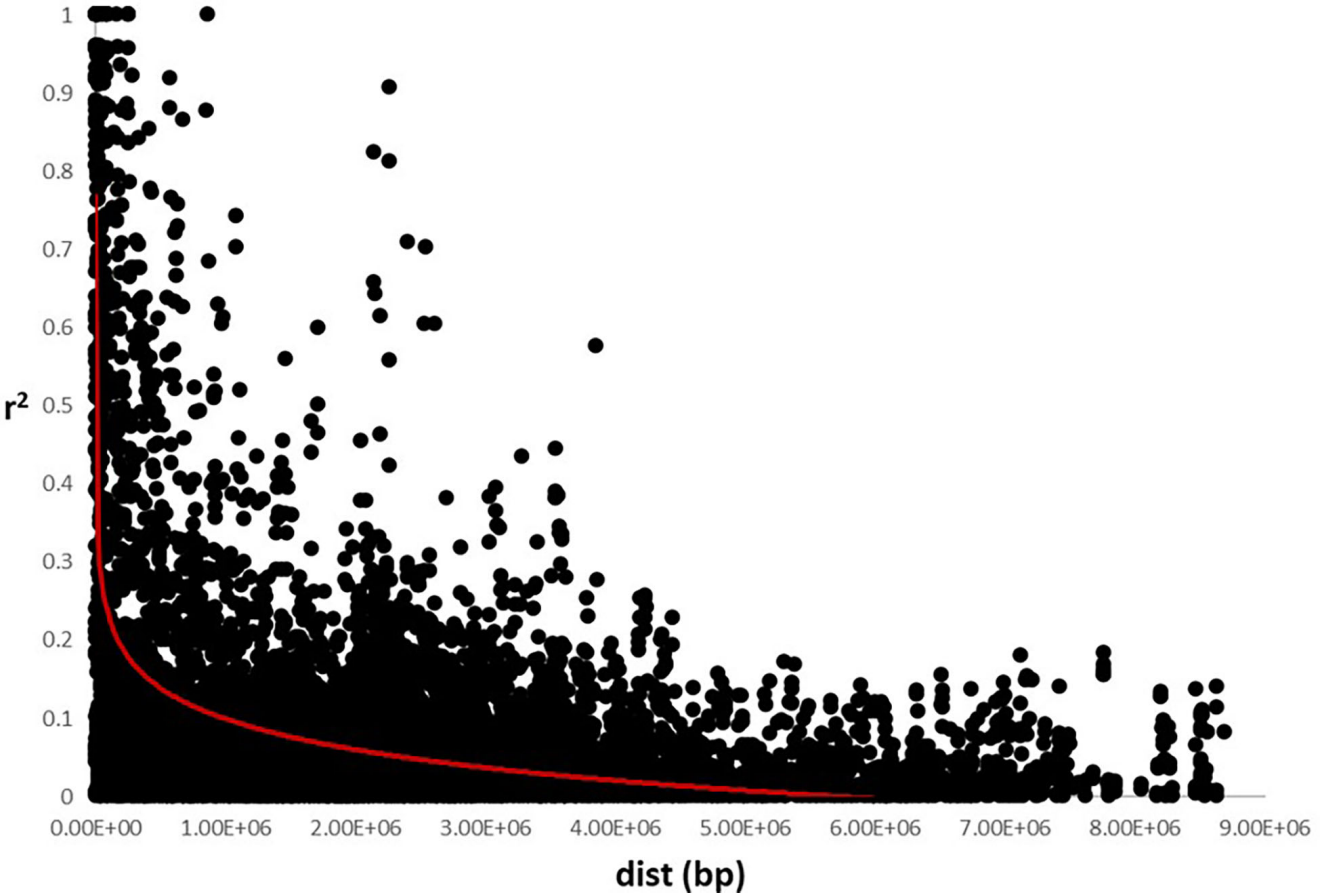
Linkage disequilibrium decay using SNP markers data set. Estimation of r^2^ versus distance in base pair (bp) was represented. LD decay was established around 0.25e^6^ bp.

The analysis of the genetic structure of the germplasm set suggested the existence of two main groups, as indicated by a K=2 using the Delta K method. LD was calculated using all the SNP markers and the LD decay was 0.25 x10^6^ bp for all the chromosomes (**Figure 2**), which is consistent with other studies in which it was observed that the linkage disequilibrium rapidly decays in sunflower (Liu and Burke, 2006; Kolkman et al., 2007; Fusari et al., 2008).

### 3.3. Marker trait association

**Figure 3:**
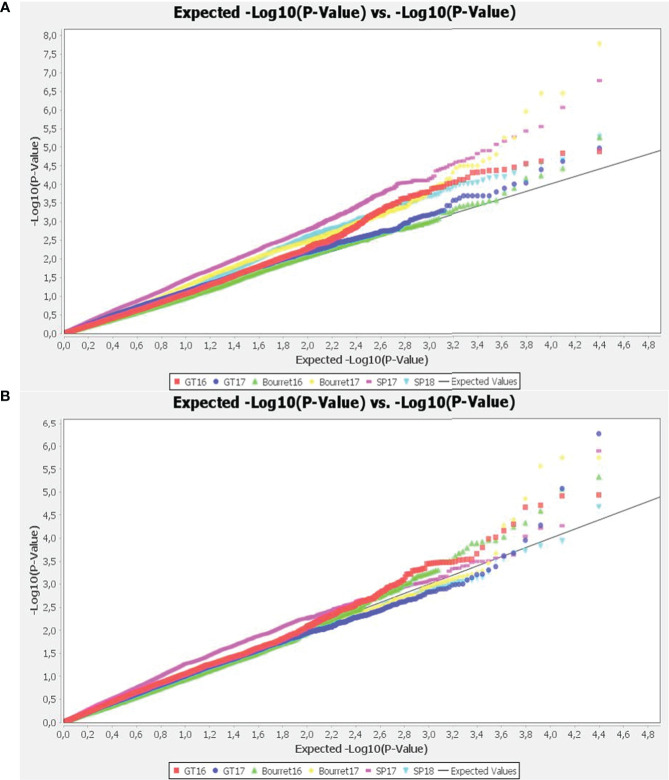
Quantile-quantile (Q-Q) plots of observed versus expected P values of the GWAS results using GLM+PCA **(A)** and MLM+K+PCA **(B)**. The straight line represents concordance of observed and expected values.

The best fitting models were chosen analysing the quantile-quantile plots (QQ-plots). The deviation of observed vs expected –log_10_ p-values was smaller for the mixed linear models (MLM) than for general linear models (GLM), with the best results within each group using GLM+PCA and MLM+K+PCA combinations with optimum level of compression and re-estimation after each marker (**Figure 3**).

**Figure 4:**
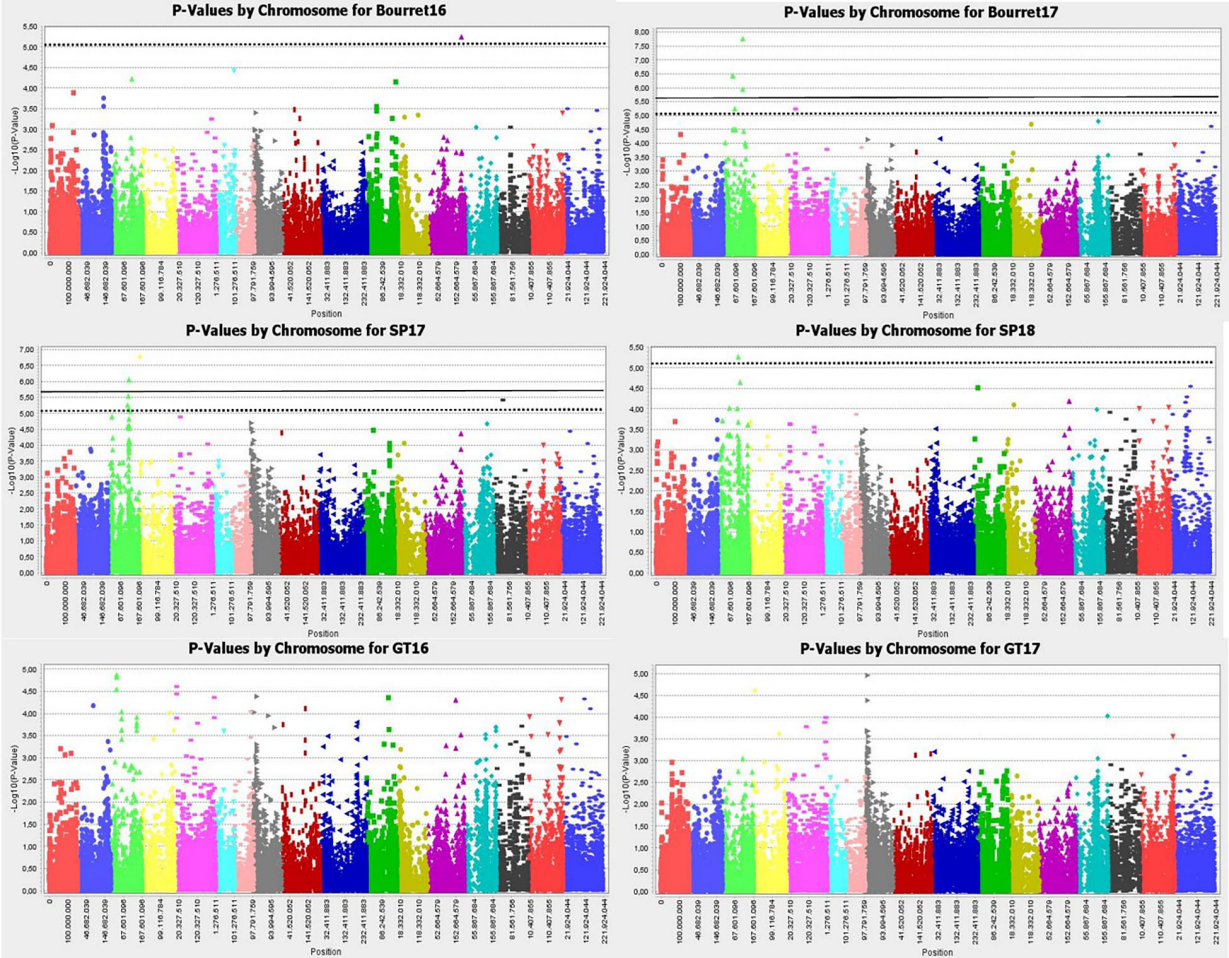
Manhattan-plots illustrating significant associations for resistance to three broomrape populations (SP, Bourret and GT) in a panel of 104 sunflower accessions evaluated in two environments each using GLM+PCA. The P values were adjusted using the Bonferroni threshold and false detection rate (FDR) correction (5% and 20%) to reduce false positive associations. The solid line corresponds to the 5% threshold and the dotted line to the 20% threshold. The vertical axis indicates –log10 of p-value and horizontal axis indicates chromosomes and physical positions of SNPs.

**Figure 5:**
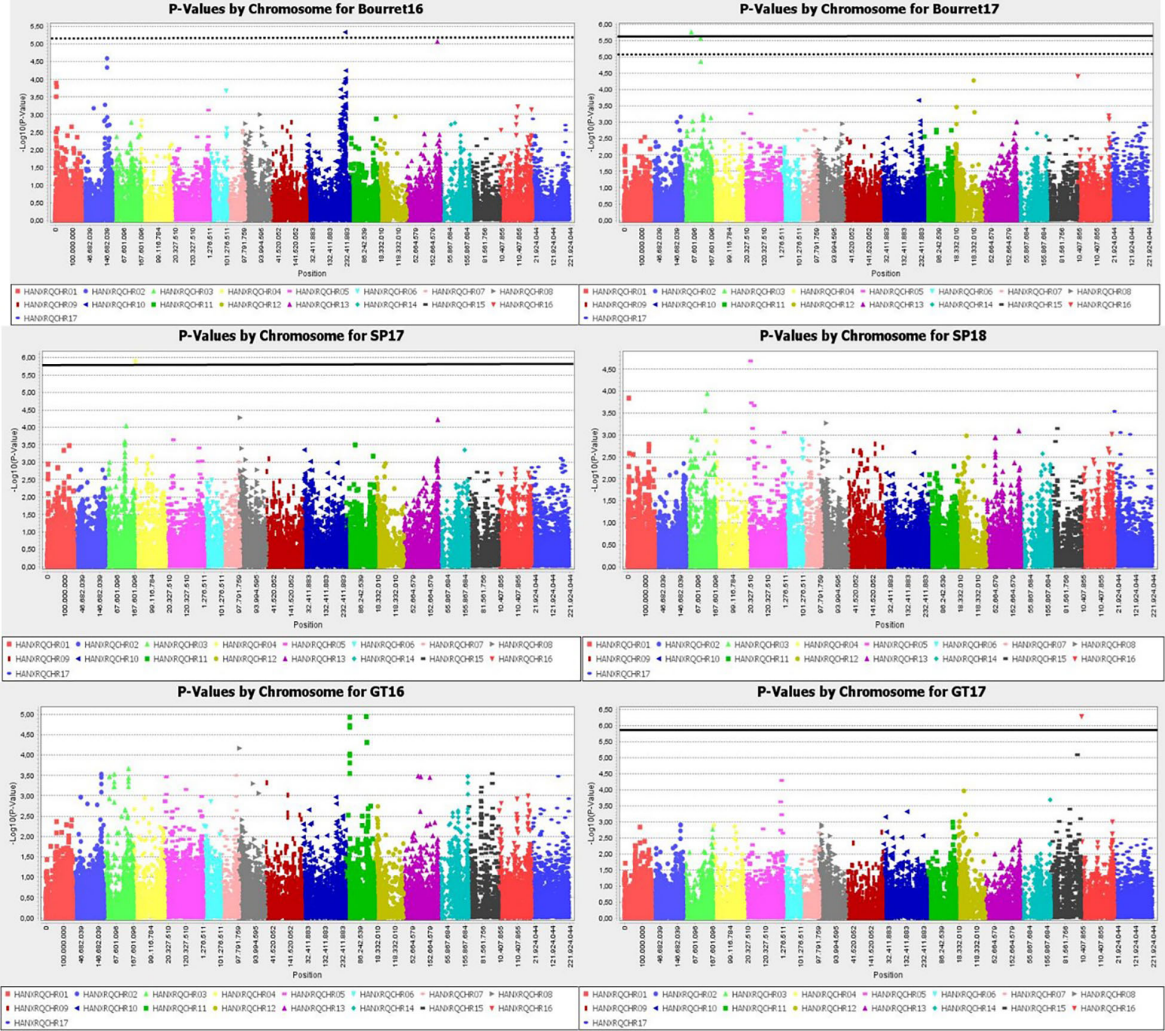
Manhattan-plots illustrating significant associations for resistance to three broomrape populations (SP, Bourret and GT) in a panel of 104 sunflower accessions evaluated in two environments each using MLM+K+PCA. The P values were adjusted using the Bonferroni threshold and false detection rate (FDR) correction (5% and 20%) to reduce false positive associations. The solid line corresponds to the 5% threshold and the dotted line to the 20% threshold. The vertical axis indicates –log10 of p-value and horizontal axis indicates chromosomes and physical positions of SNPs.

Manhattan plot for GLM+PCA (**Figure 4**) and MLM+K+PCA (**Figure 5**) revealed a total of 14 SNP markers significantly associated with the number of emerged broomrapes. There were six of them in GLM+PCA and four of them in MLM+K+PCA with p < 2E-06 (5%) and 5 additional in GLM+PCA and 2 in MLM+K+PCA with p < 8E-06 (20%) (**Table 5**). Significant associations were identified on six different chromosomes from the HanXRQr2.0-SUNRISE reference sunflower genome assembly (https://www.heliagene.org/HanXRQr2.0-SUNRISE) (**Table 5**), and for most of the broomrape populations and environments, except for GT16. Some markers were significant for two different broomrape populations and/or environments: AX-105943713 for Bourret17 and SP17, and AX-105776042 for SP17 and SP18 (**Table 5**). The trait variation explained by each marker varied from 14 to 24% (**Table 5**). The most significant peaks detected above the 5% Bonferroni threshold and identified both with GLM+PCA and MLM+K+PCA were observed on chr 3, which, in addition, showed by far the highest number of marker-trait associations. Two regions which contained clustered associations were observed on this chromosome. The first one was a 5.2 Mbp region spanned by the two markers AX-105943713 and AX-147199586 [coordinates 85486771-90700620 (HanXRQr2.0-SUNRISE)] and associated to both race E_FR_ (Bourret17) and race F_GV_ (SP17) of broomrape. The second one with markers AX-105705204, AX-105776042, AX-105655280, and AX-105768536 ranged from physical position 129889814 bp to 136591650 bp (6.7 Mbp) (HanXRQr2.0-SUNRISE) and it was associated only to broomrape race F_GV_ (SP17 and SP18) (**Table 5**). Other significant single marker-trait associations were identified on chromosomes 5, 10 and 13 for race E_FR_ populations (Bourret17 for chr 5, and Bourret16 for chr 10 and 13), chr15 for race F_GV_ population SP17, and chr16 for race G_TK_ population GT17 (**Table 5**).

**Table 5:** SNP markers associated with broomrape resistance in different environments (composed of three races evaluated over 2 years) according the GLM corrected with PCA and MLM corrected with kinship (K-matrix) and PCA.

	Trait	Marker	Chr(HanXRQr2.0)	Position (bp)(HanXRQr2.0)	Initial mapped position (bp)in HanXRQr1.0	p-value	marker_R2
GLM+PCA							
	Bourret17	AX-105943713	3	85486771	105501538	1.13E-06	0.16272
	SP17	AX-105943713	3	85486771	105501538	5.48E-06	0.14168
	Bourret17	AX-105531030	3	85505366	105482945	1.72E-08	0.23265
	Bourret17	AX-105925988	3	86489659	51278297	3.73E-07	0.19847
	Bourret17	AX-105709192	3	87263090	51917942	3.73E-07	0.19847
	Bourret17	AX-147199586	3	90700620	62576510	5.79E-06	0.14342
	SP17	AX-105705204	3	129889814	4872459	1.70E-07	0.20398
	SP17	AX-105776042	3	133359785	110766840	2.89E-06	0.1716
	SP18	AX-105776042	3	133359785	110766840	5.43E-06	0.15702
	SP17	AX-105655280	3	135970618	113178523	8.84E-07	0.18535
	SP17	AX-105768536	3	136591650	113484242	7.08E-06	0.16095
	Bourret17	AX-105759358	5	24325063	44462658	5.82E-06	0.16604
	Bourret16	AX-105929368	13	157389651	178662062	5.70E-06	0.18655
	SP17	AX-105876346	15	38533277	49324811	3.79E-06	0.16838
MLM+K+PCA							
	Bourret17	AX-105531030	3	85505363	105482945	2.70E-06	0.21737
	Bourret17	AX-105925988	3	86489659	51278297	1.76E-06	0.22416
	Bourret17	AX-105709192	3	87263090	51917942	1.76E-06	0.22416
	SP17	AX-105705204	3	129889814	4872459	1.25E-06	0.23529
	Bourret16	AX-105891155	10	167173148	221096919	4.60E-06	0.20456
	GT17	AX-105925592	16	2751833	4266574	5.32E-07	0.24252

Unshaded data corresponds to the markers that exceed Bonferroni 5% threshold and shaded markers corresponds with Bonferroni 20%.

### 3.4. Candidate genes

As mentioned above, significant associations were identified on linkage groups 3, 5, 10, 13, 15 and 16. The most relevant ones were found on two regions of chromosome 3. The first region on chr 3 was 5.2 Mbp long, delimited by markers AX-105943713 and AX-147199586. If the significant SNP was unique, the candidate gene analysis was centred on those genes containing the SNP and those found around +/- 250 Kbp. If a group of significant SNPs were found clustered, we focused on those genes containing the SNPs and on those found in the interval delimited by the tightly linked clustered SNPs (+/- 250 Kbp). Therefore, the five SNP markers in the 5.2 Mbp region were analysed as follows: (i) AX-105943713/AX-105531030, and AX-105925988/AX-105709192 as two clusters (and their +/- 250 Kbp window), and (ii) AX-147199586 as a single marker and its +/- 250 Kbp window. SNP markers AX-105943713 and AX-105531030 delimited a 18592 bp long area (coordinates from 85486771 to 85505363). Both AX-105943713 and AX-105531030 were found within a putative SWEET sugar transporter (HanXRQr2_Chr03g0103911 and HanXRQr2_Chr03g0103941, respectively) (**Table 6** and **Table S2**). Two additional coding regions in this interval were found, corresponding to putative mitochondrial carrier domain protein (HanXRQr2_Chr03g0103921) and to a putative potassium channel, voltage dependent EAG/ELK/ERG (HanXRQChr03g0075331). Very close to this interval (+/- 250Kbp) two putative transcription factors of the C3H (HanXRQr2_Chr03g0103891) and the AS2-LOB (HanXRQr2_Chr03g0103951) families were identified (**Table 6** and **Table S2**). The second area in the 5.2 Mbp interval of chr 3 showing a cluster of associations was 773431 bp long. It was flanked by SNP markers AX-105925988 and AX-105709192 (coordinates from 86489659 to 87263090), which were not found within a protein coding gene. The AX-105925988 and AX-105709192 (+/- 250000 bp) interval contained 17 protein coding regions (**Table S2**), three of them (HanXRQr2_Chr03g0104061, HanXRQr2_Chr03g0104071, and HanXRQr2_Chr03g0104081) corresponded to putative geraniol 8-hydroxylases (cytochrome P450 genes). Annotation of two of these cytochrome P450 coding regions was corrected in NCBI *Helianthus annuus* Annotation Release 101 and they were grouped as one single locus coding for a 7-ethoxycoumarin O-deethylase (LOC110929042), which was confirmed through Blast searches of its genomic and RNAs sequences. Also, a putative non-specific serine/threonine protein kinase was identified in this interval (HanXRQr2_Chr03g0104051) (**Table 6** and **Table S2**). Finally, within the 5.2 Mbp region, single marker trait association for race E_FR_ (Bourret17) for marker AX-147199586 (position 90700620) was analysed for candidate genes. This SNP was found within a putative R-linalool synthase (HanXRQr2_Chr03g0104971). The AX-147199586 (+/- 250000 bp) area showed three protein coding genes, and among them a putative transcription factor interactor and regulator of the CCHC(Zn) family (HanXRQr2_Chr03g0104961) was identified. The above described candidate genes were those tightly linked to the significant SNPs; however, it is worth mentioning that exploration of the 5.2 Mbp region in the the AX-105925988/AX-105709192 to AX-147199586 interval outside the areas already described revealed an important proportion of protein kinase genes [out of 57 protein coding genes annotated in this region, 12 (21%) of them corresponding to protein kinases of the RLK-Pelle-LRR-I-1 f, RLK-Pelle-LRR-VIII-1, RLK-Pelle-LRR-XI-1, RLK-Pelle-CR4L, RLK-Pelle-SD-2b, RLK-Pelle-WAK, CMGC-GSK, and CMGC-CDK-CRK7-CDK9 families] (**Table 6** and **Table S2**).

**Table 6:** Summary of the most relevant genes identified as associated to significant marker trait associations (detailed information in **Table S2**).

Chr	Interval explored	N. genes^a^	SNP markers (clustered or single-marker associations)	SNP position (bp) (HanXRQr2.0)	Population	Most relevant genes^b^	Gene start position (bp) (HanXRQr2.0)	Description
						HanXRQr2_Chr03g0103891	85268543	Putative transcription factor C3H family
3	AX-105943713 to AX-105531030 and their 250 Kbp window	11	AX-105943713	85486771	Bourret17/SP17	HanXRQr2_Chr03g0103911	85462527	Putative SWEET sugar transporter
						HanXRQr2_Chr03g0103921	85489385	Putative mitochondrial carrier domain protein
						HanXRQr2_Chr03g0103931	85489947	Putative potassium channel, voltage-dependent, EAG/ELK/ERG
			AX-105531030	85505366	Bourret17	HanXRQr2_Chr03g0103941	85501458	Putative SWEET sugar transporter [bidirectional sugar transporter SWEET17 (LOC110929598)]
						HanXRQr2_Chr03g0103951	85631556	Putative transcription factor AS2-LOB family
3	AX-105925988 to AX-105709192 and their 250 Kbp window	17				HanXRQr2_Chr03g0104051	86256736	Putative non-specific serine/threonine protein kinase
						HanXRQr2_Chr03g0104061	86283258	Putative geraniol 8-hydroxylase
						HanXRQr2_Chr03g0104071	86285554	Putative geraniol 8-hydroxylase [7-ethoxycoumarin O-deethylase (LOC110929042)]
						HanXRQr2_Chr03g0104081	86328955	Putative geraniol 8-hydroxylase [7-ethoxycoumarin O-deethylase (LOC110929042)]
			AX-105925988	86489659	Bourret17			
			AX-105709192	87263090	Bourret17			
3	Bigger interval from AX-105925988/AX-105709192 to AX-147199586 outside their 250 Kbp window	57						12 protein kinase genes (Four MDIS1-interacting receptor like kinase 2; one receptor-like protein kinase ANXUR1; one of the CMGC-CDK-CRK7-CDK9 family; one of the RLK-Pelle-SD-2b family; two of the RLK-Pelle-CR4L family, one of the RLK-Pelle-LRR-I-1 family, one of the RLK-Pelle-WAK family, and one of the CAMK-CDPK family)
3	AX-147199586 and its 250 Kbp window	3				HanXRQr2_Chr03g0104961	90567938	Putative transcription factor interactor and regulator CCHC(Zn) family
			AX-147199586	90700620	Bourret17	HanXRQr2_Chr03g0104971	90699125	Putative R-linalool synthase
3	AX-105705204 and its 250 Kbp window	22				HanXRQr2_Chr03g0116041	129875663	Putative transcription factor interactor and regulator CCHC(Zn) family
						HanXRQr2_Chr03g0116071	129888590	Putative transcription factor TFIIIC, triple barrel domain-containing protein
			AX-105705204	129889814	SP17			
3	AX-105776042 and its 500 Kbp window							One Putative protein kinase RLK-Pelle-LRR-I-2 family (HanXRQr2_Chr03g0116831), and three putative transcription factors of the C2H2 family (HanXRQr2_Chr03g0116871), of the Hap3/NF-YB family (HanXRQr2_Chr03g0116881), and of the CCHC(Zn) family (HanXRQr2_Chr03g0116891)
			AX-105776042	133359785	SP17			
3	AX-105655280 to AX-105768536 and its 250 Kbp window	35				HanXRQr2_Chr03g0117711	135874296	Putative mitogen-activated protein kinase STE-STE11 family
			AX-105655280	135970618	SP17	HanXRQr2_Chr03g0117741	135961367	Putative 1,4-alpha-glucan branching enzyme
			AX-105768536	136591650	SP17			
5	AX-105759358 and its 250 Kbp window	8				HanXRQr2_Chr05g0200451	24028576	Putative transcription factor interactor and regulator CCHC(Zn) family
			AX-105759358	24325063	Bourret17			
10	AX-105891155 and its 250 Kbp window	20				HanXRQr2_Chr10g0458681	167025903	Putative transcription factor TIFY family [Protein TIFY 10c (LOC110886429)]
			AX-105891155	167173148	Bourret16			
						HanXRQr2_Chr10g0458741	167193456	Putative transcription factor MYB-related family
						HanXRQr2_Chr10g0458761	167211637	Putative transcription factor MYB family [Transcription factor MYB3 (LOC110883374)]
13	AX-105929368 and its 250 Kbp window	32				HanXRQr2_Chr13g0610991 HanXRQr2_Chr13g0611011 HanXRQr2_Chr13g0611021	157302732; 157322524, 157325775	Three putative non-specific serine/threonine protein kinase genes
						HanXRQr2_Chr13g0611031	157334169	Putative disease resistance RPP13-like protein 1 (LOC110902132)
						HanXRQr2_Chr13g0611041	157358197	Putative protein kinase CK1-CK1 family
			AX-105929368	157389651	Bourret16	HanXRQr2_Chr13g0611081	157386420	Putative splicing factor 3B subunit 5/RDS3 complex subunit 10
						HanXRQr2_Chr13g0611091	157419694	Putative cytochrome P450 [Alkane hydroxylase MAH1 (LOC110899957)]
						HanXRQr2_Chr13g0611131	157482837	Putative cytochrome P450 [Alkane hydroxylase MAH1 (LOC110899958)]
15	AX-105876346 and its 250 Kbp window	23				HanXRQr2_Chr15g0687981	38520847	Putative protein kinase RLK-Pelle-DLSV family
			AX-105876346	38533277	SP17			
						HanXRQr2_Chr15g0688011	38600086	Putative transcription factor of the C2H2 family
						HanXRQr2_Chr16g0724281	2553825	Putative transcription factor bHLH family
16	AX-105925592 and its 250 Kbp window	23	AX-105925592	2751833	GT17	HanXRQr2_Chr16g0724391	2748783	Putative RNA recognition motif domain, mei2/Mei2-like RNA recognition [Protein MEI2-like 1 (LOC110914999)]
						HanXRQr2_Chr16g0724411	2760903	Putative transcription factor AP2-EREBP family [Ethylene-responsive transcription factor ERF114 (LOC110917506)]
						HanXRQr2_Chr16g0724481, HanXRQr2_Chr16g0724491, HanXRQr2_Chr16g0724511, HanXRQr2_Chr16g0724531	2847073, 2902831, 2945156 , 2982242	Four putative chromatin regulators of the PHD family

^a^Number of annotated genes in the specified interval.

^b^Most relevant genes (i) containing the SNP (shaded); (ii) in the interval spanned by clustered markers, within a 250 Kb window, or (iii) closely located but outside the 250 Kbp window.

The second region on chr 3 showing a cluster of associations ranged from physical positions 129889814 bp to 136591650 bp (6.7 Mbp long). Due to the distance found between the four SNP markers in this interval, AX-105705204 and AX-105776042 were analysed as single markers and their +/- 250Kbp window, and AX-105655280, and AX-105768536 as an interval and their +/- 250Kbp window. The AX-105705204 +/- 250Kbp window contained 22 protein coding genes (**Table 6** **Table S2**). Among them, a putative transcription factor interactor and regulator of the CCHC (Zn) family (HanXRQr2_Chr03g0116041) and a putative transcription factor TFIIIC (HanXRQr2_Chr03g0116071) were found tightly linked to this SNP. The AX-105776042 +/- 250Kbp region had 9 annotated genes (**Table S2**), which were not associated with plant resistance to pathogens. However, when exploring a larger window of 500 Kbp, three tightly linked transcription factors of the C2H2, Hap3/NF-YB, and CCHC(Zn) families were found 300 Kbp upstream this region, and other three of the CCHC(Zn) and C2H2 families were identified 500 Kbp downstream the abovementioned region (**Table 6** **Table S2**). Finally, within the AX-105655280 to AX-105768536 interval (+/- 250Kbp) a putative mitogen-activated protein kinase of the STE-STE11 family was identified (**Table 6**, **Table S2**).

Exploration of the genomic region surrounding the unique markers (+/- 250Kbp) of the remaining chromosomes revealed close genes that included proteins that might be associated with disease resistance, such as a putative transcription factor interactor and regulator of the CCHC(Zn) family (HanXRQr2_Chr05g0200451) in chr 5; three putative transcription factors of the TIFY and MYB families (HanXRQr2_Chr10g0458681, HanXRQr2_Chr10g0458741, HanXRQr2_Chr10g0458761) in chr 10; five protein kinases, a putative virus X resistance protein-like, two putative transcription factor interactor and regulator of the CCHC(Zn) family and five putative cytochrome P450s (two of them renamed as alkane hydroxylases MAH1) in chr 13; a putative protein kinase of the RLK-Pelle-DLSV and a putative transcription factor of the C2H2 family in chr 15; and two putative transcription factors (of the bHLH and AP2-EREBP families) and four clustered putative chromatin regulators of the PHD family in chr 16 (**Table 6**; **Table S2**).

## 4. Discussion

Resistance to broomrape in commercial sunflower hybrids is mainly qualitative, controlled by dominant alleles at major genes. However, this type of resistance is easily surpassed by the parasite, leading to a continuous race evolution that makes it difficult the control of the parasite by means of genetic resistance (Fernández-Martínez et al., 2015). Alternative sources of resistance, such as those under quantitative genetic control are required. To that end, genome-wide association study (GWAS) is an optimized approach to identify new genes associated with resistance to broomrape in sunflower. Using GLM and MLM analysis, in combination with kinship and principal component analysis (PCA), which reduce the computation demand and solve the problems related to type I and type II error rates (Yu et al., 2006), a total of 14 single nucleotide polymorphisms (SNP) significantly associated with resistance to sunflower broomrape were identified. Although complete resistance has been found in the sunflower set for the three broomrape populations, the evaluated trait NEBS showed mostly a continuous distribution in the accessions. Considering this, the number of genomic regions identified and their minor effects, this study confirmed the involvement of quantitative resistance mechanisms in genetic resistance to broomrape in sunflower, controlled by multiple minor QTL associated to the number of broomrape shoots per plant, as has been described previously (Pérez-Vich et al., 2004; Akhtouch et al., 2016; Louarn et al., 2016; Imerovski et al., 2019; Baytar et al., 2021). The complementary use of major genes with resistance mechanisms under quantitative genetic control has been proposed as an approach for developing more durable genetic resistant to sunflower broomrape (Pérez-Vich et al., 2013). A greater durability of such polygenic resistance compared to monogenic resistance has been demonstrated in other pathosystems involving viruses, fungi, and nematodes (Palloix et al., 2009; Brun et al., 2010; Fournet et al., 2013).

Research on the development of resistance sources to broomrape in sunflower has been mainly focused on vertical mechanisms of resistance controlled by single loci with a major effect (Imerovski et al., 2019). Contrarily, little efforts have been devoted to the identification of germplasm with quantitative resistance to broomrape, with few accessions currently available for genetic studies. This fact has limited the size of the GWAS population used in the present study, consisting of 104 accessions but including 34 accessions for which quantitative resistance had been observed previously. Additionally, the accessions were tested with three contrasting broomrape populations under two environments, in all cases using artificial inoculation. To the best of our knowledge, there are no previous studies on the analysis of resistance to broomrape (*Orobanche* spp. and *Phelipanche* spp.) in sunflower or other crop species and accordingly it is not possible to compare the present study with previous ones. For resistance to other parasitic plants, e.g. *Striga* spp. in maize and sorghum, most association mapping studies were based on population sizes not very far from our population size, e.g. n=132 (Adewale et al., 2020), n=150 (Stanley et al., 2021), n=169 (Okunola et al., 2022), or n=173 (Kavuloko et al., 2021), in most cases using a single *Striga* population. Other studies used larger population sizes, e.g. n=380 (Gowda et al., 2021).

Most of the significant markers found in this study were located in two different regions of chr 3. The upper region was associated to both race E_FR_ (Bourret17) and race F_GV_ (SP17). It was 5.2 Mbp long and spanned from 85.5 to 90.7 Mbp (HanXRQr2.0-SUNRISE), delimited by markers AX-105943713 and AX-147199586. The lower one ranged from physical positions 129.8.0 Mbp to 136.6 Mbp (6.7 Mbp) (HanXRQr2.0-SUNRISE). It was delimited by AX-105705204 and AX-105768536 markers and associated only to race F_GV_ resistance (SP17 and SP18). In chr 3, genes conferring resistance to sunflower broomrape have been reported. Thus, Tang et al. (2003) and Pérez-Vich et al. (2004) identified the gene *Or5*, conferring resistance to sunflower broomrape race E, on the upper telomeric region of this chromosome. These mapping studies located this gene to the end of chr 3 distal to the SSR marker locus CRT392 (Tang et al., 2003), which is the uppermost SSR on chr 3, and to the RFLP marker locus ZVG406 (Pérez-Vich et al., 2004), which is the uppermost RFLP on chr 3, and co-segregating in BSA with the TRAP marker TRC27133 based on chr 3 telomeric sequences (Márquez-Lema et al., 2008). CRT392 is tightly linked to the SFW8304 SNP marker locus (Bowers et al., 2012), located at 5.2 Mbp (HanXRQr2.0-SUNRISE). Also, although the effect of the *Or5* major gene on the number of broomrapes per plant could be detected in an about 15 cM support interval, it was clearly centred on the chr 3 telomeric region (Pérez-Vich et al., 2004). Considering this and the position of the significant SNP on chr 3 from this study, it seems likely that the significant regions detected on this chromosome do not underlie the major gene *Or5*, and their effects are more related to other mechanisms associated to a quantitative component of broomrape resistance. In addition to *Or5*, Imerovski et al. (2019) identified two regions also in chr 3 associated with broomrape resistance: the region between 31.97 and 38.48 Mbp (from HanXRQr1.0 assembly), named as QTL *or3.1* by the authors, and the region between 97.13 and 100.85 Mbp (from HanXRQr1.0 assembly), named QTL *or3.2*. These locations are close to the two significant regions on chr 3 identified in this study [the upper and lower chr 3 regions showed significant SNPs at positions 51.3 to 51.9 and 110 to 113 Mbp, respectively in the HanXRQr1.0 assembly (**Table 5**)]. The study of Imerovski et al. (2019) was based on bi-parent genetic populations and therefore analytical and experimental procedures were completely different to those used in this study. In addition, these authors used only one broomrape population, race G from Serbia, also different to those used in this research. The fact that two different significant regions on chr 3 have also been identified in this study, at close proximity to the *or3.1* and *or3.2* QTL intervals defined by Imerovski et al. (2019), indicates that both QTL are likely to be coincident with the two regions detected on chr 3 in this study, and that they are stable and expressed over a wide range of environments, analytical procedures and broomrape races. Additionally, Akhtouch et al. (2016) identified a QTL on chr 3 associated with recessive resistance to race F_GV_ of broomrape in line K-96, flanked by SSR markers ORS338 and ORS10. ORS338 blast searches against the HanXRQr2.0-SUNRISE assembly located this marker at 138.7 Mbp, very close to the lower chr 3 significant 6.7 Mbp interval from this study. The present study reinforces therefore the importance and effect of chr 3 regions, other than the major *Or5* gene, on broomrape resistance in sunflower.

Following the same nomenclature of Imerovski et al. (2019), the *or3.1* region in our study was that spanning from 85.5 to 90.7 Mbp (HanXRQr2.0-SUNRISE). Within this *or3.1*–5.2 Mbp region, candidate genes were identified at or tightly linked to the significant SNPs in three separate intervals containing 11, 17 and 3 protein coding genes, associated with Bourret17/SP17, Bourret17, and Bourret17, respectively. Among these candidate genes, two of them carrying the SNPs AX-105943713 and AX-105531030 were SWEET (Sugars Will Eventually be Exported Transporters) sugar transporter genes. SWEET transporters are mainly involved in the efflux of both mono- and di-saccharides from the site of synthesis to the sink organs, like grains, flowers, or roots (Chen et al., 2010; Chen et al., 2012). They play a critical role in important plant physiological processes such as pollen nutrition, nectar secretion, stress tolerance, phloem transport, and plant-microbe interactions (Jeena et al., 2019). It has been shown that pathogens use these genes to extract sugars for their nutrition, and that SWEET genes are negative regulators of disease resistance (Devanna et al., 2021). One of the best-known examples of this is the *Xa13* (SWEET11/Os8N3) locus in rice which is responsible for recessive resistance to blight caused by *Xanthomonas oryzae* pv. *oryzae*. In this case, the pathogen effector molecule TAL (transcription activator-like) precisely binds with a *cis* regulatory element of the SWEET11 gene promoter and modulates its transcription for enhancing the efflux of sugars which are utilized by the pathogen. The loss of pathogen-induced transcriptional motivation alters the plant-pathogen reaction from susceptibility to resistance (Jeena et al., 2019; Devanna et al., 2021). In the context of a plant-to-plant parasitic relationship, sucrose transfer at the host-parasite interface, in addition to sucrose phloem unloading in the sink tissues of tubercle and shoot, represent key processes in the parasite growth (Misra et al., 2019). There are no previous reports on the role of SWEET genes in resistance to parasitic plants, but their involvement in parasite development and sunflower resistance deserves further studies. In addition to SWEET genes, the *or3.1*–*Or5*-5.2 Mbp region showed a putative non-specific serine/threonine protein kinase tightly linked to SNP AX-105925988, as well as 12 protein kinase genes. So far, the only gene conferring resistance to sunflower broomrape that has been cloned in sunflower (*Or7* on chr 7) has been identified as a receptor-like protein kinase gene (Duriez et al., 2019). Accordingly, the kinase genes identified as candidate genes in this research are promising candidates for future investigations.

The *or3.2* region on chr 3 associated to race G resistance reported by Imerovski et al. (2019) was likely to be coincident with that delimited by AX-105705204 and AX-105768536 in the present study, which ranged from physical positions 129.8.0 Mbp to 136.6 bp (6.7 Mbp) (HanXRQr2.0-SUNRISE). In our study, markers from that region were associated with broomrape resistance to race F_GV_ (SP17 and SP18), but not with resistance to race G_TK_ (**Table 5**). Within the AX-105705204 and AX-105768536 interval, transcription factors of several families were tightly linked to the significant SNPs. Plant transcription factors play roles in diverse biological processes, including defensive responses to pathogens, in which they regulate genes related to pathogen-associated molecular pattern-triggered immunity, effector-triggered immunity, hormone signalling pathways, and phytoalexin biosynthesis (Seo and Choi, 2015). Also, they have been reported to be hubs targeted by multiple pathogen effectors in diverse ways (Mukhtar et al., 2011). Yang et al. (2020) showed that genes related to transcription factors were highly induced in a resistant sunflower cultivar after inoculation with a broomrape population of race G from China, while more transcription factor genes were found down-regulated than up-regulated in a susceptible cultivar.

For resistance to the Bourret population (race E_FR_), chromosomes 5, 10, 13 also showed significant marker-trait associations. Again, transcription factors and protein kinase genes were found tightly linked to the significant SNPs at these regions. Pérez Vich et al., (2004); Akhtouch et al. (2016); Louarn et al. (2016) and Imerovski et al. (2019) also found QTL in these chromosomes associated to races E, F or G using bi-parent populations. Particularly, the QTL on chr 13 *or13.2* was located by Imerovski et al. (2019) at about 174.8 Mbp (HanXRQr1.0), associated to race G from Serbia and by Pérez Vich et al. (2004) close to the RFLP marker locus ZVG547, located at 173.1 Mbp (HanXRQr2.0), and associated to race F_GV_. These positions are close to the significant chr 13 SNP [AX-105929368; 157.4 Mbp (HanXRQr2.0), 178.7 Mbp (HanXRQr1.0)] detected in this study. Additionally, two QTL for resistance to populations SP (race F_GV_) and GT (race G_TK_), respectively, were detected at chromosomes 15 and 16. For race G_TK_, this was the only significant marker-trait association found, which differed from results found for the other two races evaluated. The effect of a quantitative component determining partial resistance has been described for a race G population from Serbia (Imerovski et al., 2019). A putative ethylene responsive transcription factor ERF114 (AP2-ERF) was found tightly linked (at 9070 bp) to the significant SNP on chr 16. Interestingly, in the interaction between the parasitic weed *Striga hermonthica* and rice, the systemic-acquired resistance (SAR) pathway is regulated by both jasmonic acid (JA) and salycilic acid (SA) in a cross talk mediated by WRKY45 (Mutuku et al., 2015) and regulated by ethylene responsive factor (AP2/ERF) transcription factor (Licausi et al., 2013). Additionally, AP2/ERFs were found to be significantly associated with *S. hermonthica* resistance to maize in a GWAS study (Adewale et al., 2020).

In sunflower, several GWAS studies have been conducted on traits such as basal and apical branching (Nambeesan et al., 2015), abiotic stresses (Mangin et al., 2017), flowering time (Bonnafous et al., 2018) or flower morphological traits (Dowell et al., 2019). However, there are no previous studies on the use of GWAS approach to analyse resistance to sunflower broomrape. The present study, using three broomrape populations with contrasting degree of virulence, revealed several genomic regions that were associated with broomrape resistance. Candidate genes putatively involved in broomrape resistance were identified in these regions. This information will serve as a basis for the identification and characterization of novel broomrape resistance genes of value for developing durable genetic resistance to this parasitic weed.

## Data availability statement

The original contributions presented in the study are included in the article/**Supplementary Material**. Further inquiries can be directed to the corresponding authors.

## Author contributions

SM and BP-V conceived the work and planned and supervised the research. NP conducted plant genotyping. AC-G, JL, MCB and LV conducted phenotypic evaluations. AC-G, SM, and LV conducted statistical analyses. AC-G, LV, and BP-V wrote the draft of the manuscript. All authors contributed to the article and approved the submitted version.
